# Virtual reality experience in physical education and sports lessons: An investigation into its effect on the balance and coordination skills of middle school students

**DOI:** 10.1371/journal.pone.0354258

**Published:** 2026-08-12

**Authors:** Nazlı Yanar Tunçel, Züleyha Avşar

**Affiliations:** 1 Karamanoğlu Mehmetbey Universtiy, Faculty of Sports Sciences, Karaman, Türkiye; 2 Bursa Uludağ Universtiy, Faculty of Sports Science, Bursa, Türkiye; University of Salerno: Universita degli Studi di Salerno, ITALY

## Abstract

This study aims to investigate the effect of playing a virtual reality (VR) game on the balance and coordination skills of students. A total of 39 7th-grade students volunteered to participate in the study; 20 were in the experimental group and 19 were in the control group. The study used an experimental design in which participants played games with virtual reality headsets for eight weeks. The Y Balance Test and the Children’s Body Coordination Test (Körperkoordinationstest für Kinder – KTK) were used to assess balance and coordination skills before and after the intervention. The KTK is a coordination test comprising four subtests: Backward Balancing, Single-Leg Hopping, Side-To-Side Hopping, and Side-To-Side Walking on a Platform. The data were analysed using Jamovi (version 2.3.21.0) statistical software. The results of the mixed-design analysis of variance revealed significant group × time interactions in favour of the experimental group for Backward Balancing, Lateral Jumping, Platform Transport, the total KTK score and right and left Y-balance performance. However, there was no significant difference in single-leg jumping performance (p < .05). These results suggest that virtual reality-based applications could improve balance and coordination in secondary school students. Experiencing the VR game was found to significantly improve students’ balance and coordination skills. It is recommended that virtual reality-based applications be used as a supplementary teaching tool in physical education and sports lessons to support the development of motor skills.

## Introduction

The increasing use of technology in society has driven the digitisation of education [1]. Countries that value education and keep pace with technology have incorporated digital skills into the classroom as teaching and assessment tools [2]. There are also high expectations that digital technology will support student learning in schools [3]. While technology can easily be incorporated into some subjects, its inclusion in others has sparked debate. New and positive approaches to incorporating technology into specific disciplines, such as physical education and sports, should be explored [4]. Given the specific technologies used in these disciplines, it is particularly important to focus on technology in physical education and sports lessons. Using technology to develop parameters specific to physical education and sports, such as balance and endurance, can make things more convenient for teachers and educators.

Balance is one of the most important physical attributes that children need to develop. The secondary school curriculum includes a number of learning outcomes designed to develop balance, particularly for students at this level [5]. Therefore, physical education teachers should incorporate balance exercises into their lessons, particularly for secondary school students. There are various methods and techniques that can be employed to improve balance. Mobile floor equipment such as wobble/balance boards, foam rollers, swing balls and balance discs are commonly used for this purpose [6]. However, technological advances have made it possible to develop motor skills using alternative methods. One such technology is virtual reality (VR), which first emerged in the 1960s. Computer simulation systems have evolved alongside changes in imaging, multimedia, and simulation technology, enabling applications in many fields [7,8]. VR technology encompasses various methods, such as sound localisation, modelling, and spatial tracking. Furthermore, VR technology can integrate multiple methods and technologies to leverage its extraordinary advantages [9]. Through effectively simulating the natural world, VR technology can provide participants with a sufficient imaginative space and environment [10]. Furthermore, applying VR technology in the classroom can enrich teachers’ teaching tools, create a virtual environment for students, stimulate their enthusiasm, guide their imagination, increase learning efficiency and reduce teachers’ workload. VR games can provide students with multiple visual, auditory and tactile sensations, thereby enriching their learning experiences [11]. VR technology requires greater physical engagement than traditional video games. Similarly, active video games (exergames) that involve physical movement have been reported to have a positive effect on children’s levels of physical activity, balance, and motor skills [12,13]. Wang et al. [14] observed that incorporating physical activity into a sedentary video game environment could enhance student engagement and serve as an effective teaching tool in physical education classes.

Educators are attracted to VR technology due to the numerous games and activities that can be incorporated into teaching programmes for pedagogical purposes. Using VR games in school physical education classes has been shown to improve students’ overall motor coordination [15,16]. Liu et al. [17] concluded in their study that VR simulation results improve training and learning ability in physical education classes. Consequently, schools can be regarded as an alternative teaching method in physical education curricula, encouraging physical activity among children and young people. Furthermore, given that schools have the capacity to reach everyone, this is considered important in terms of encouraging physical activity among children. Technology facilitates new methods and learning environments, and increases interest in new ways of acquiring knowledge and skills at student and athlete levels [18]. The growing technology of VR is of great importance in daily life and sport. It is said to improve balance and coordination [19–22]. Players of action VR games demonstrate faster reaction times [23], improved attention [24], faster visual-motor reaction times and better balance [25]. However, it has been reported that these positive effects are largely the result of regular, repeated sessions. Single-session SG sessions may not produce significant changes in motor and cognitive performance [26].

VR has a positive impact on students’ emotional state, social interactions and learning outcomes [27]. Recent technological developments have advanced the design and use of virtual reality (VR) devices, significantly enhancing users’ motivation and active participation [28,29]. However, it is clear that most existing studies have focused on adults, clinical groups or rehabilitation applications, while those targeting educational settings are limited. In this context, there is a need for a more detailed examination of the impact of VR-based applications on balance and coordination skills in school PE lessons. This study hypothesises that VR applications will enhance students’ balance and coordination.

## Methods

### Research model

This study employed a pre-test-post-test design with experimental and control groups. In such a design, participants can be randomly assigned to groups, with one group designated as the control group and the other as the experimental group [30].

### Research group

Convenience sampling was used to select the sample for this study. This method relies on collecting data from participants who are easily accessible to the researcher, and is particularly favoured in quantitative research due to its advantages in terms of time and cost [31]. In this study, participants were therefore selected from volunteer 7th-grade students attending the same school. A total of 39 participants were included in the study: 20 in the experimental group and 19 in the control group. A sensitivity analysis was conducted using G*Power 3.1 software to evaluate the size of the study sample. Based on α = .05, power (1 − β) =.80 and a total sample size of N = 39, it was determined that the study had sufficient power to detect effect sizes of d = 0.90 and above.

**Table:** 

Inclusion criteria	Exclusion criteria
- Attending middle school (7th) - Being in good physical health (i.e., having no health issues that would impair balance and coordination skills). - Being comfortable with exposure to the VR experience. - Participation in the study is voluntary. - Parental consent having been obtained.	- Having a physical or mental disability - Feeling overly sensitive or uncomfortable with the VR experience. - Not wanting to participate in the study - Not having obtained parental consent

### Data collection tools

Two different measurement tools were used in this study to assess participants’ motor skills and balance levels. The Children’s Body Coordination Test [32] and the Y Balance Test [33].

**Fig 1:**
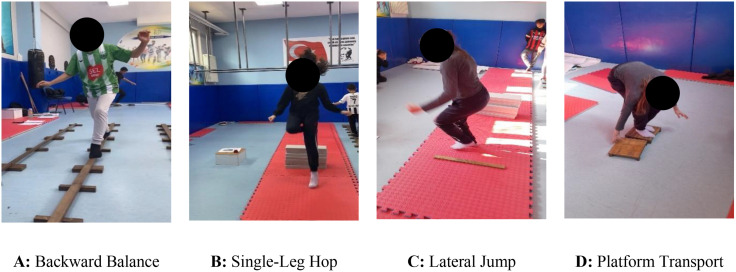
The Children’s Body Coordination Test. (The researchers took the photographs and obtained verbal consent from the parents.).

The Body Coordination Test for Children (Körperkoordinationstest für Kinder – KTK): Designed to assess the movement and coordination abilities of children aged 5–14, it was developed by Kiphard and Schilling [34] and adapted into Turkish by Özkara and Kalkavan [32]. It consists of four physical tests: Backward Balance (BB), Single-Leg Hop (SLH), Lateral Jump (LJ) and Platform Transport (PT). Each subtest is marked separately, and the total score for the KTK is obtained by adding up the marks obtained from the subtests. The average application time for each participant is 15 minutes. It can be used with all school-age children, from those with high-level motor skills to those with motor skill difficulties. The test results are categorised as follows: ‘poor motor coordination’ (MC < 56), ‘severe motor impairment’ (MC 56–70), ‘moderate motor impairment’ (MC 71–85), ‘normal’ (MC 86–115), ‘good’ (MC 116–130) and ‘very good’ (MC 131–145+). Fig 1 shows the subdimensions of the test and examples of its application.

**Fig 2:**
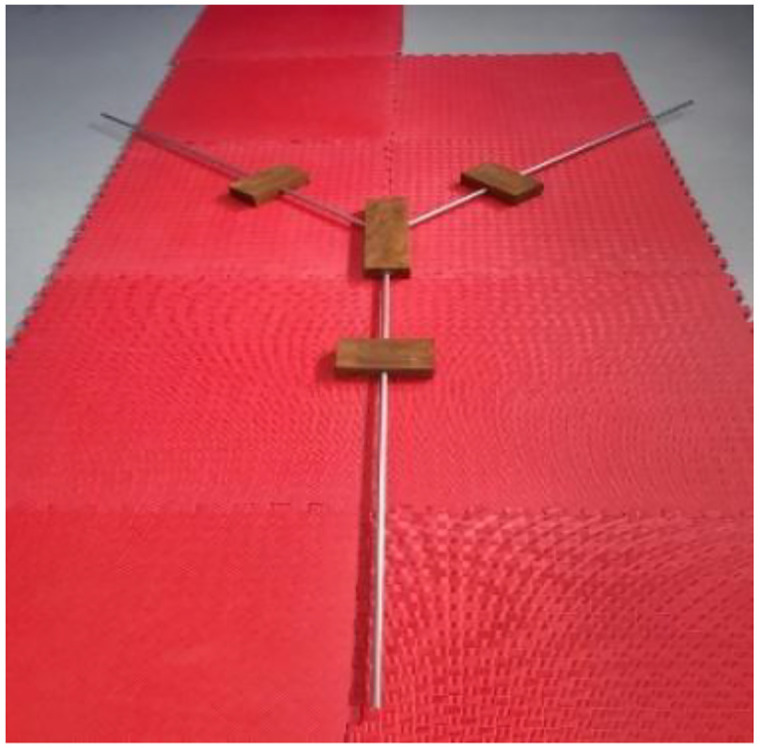
Y Balance Kit.


Balance Test: The Y Balance Test was used to measure participants’ dynamic balance. The test’s validity and reliability were determined using the Intraclass Correlation Coefficient (ICC), with an intrarater range of 0.85–0.91 and an interrater range of 0.99–1.00 [33]. After the test content and administration were explained to the students, their reach on their right and left extremities was measured using a Y Balance Kit (Fig 2) prepared according to the specifications in the literatüre. This consisted of a 35 cm long, 13 cm wide and 4 cm high wooden beam with two 2-metre-long arms extending in three directions. Participants were asked to stand on one foot at the centre of the setup and extend their toes in the anterior, posteromedial and posterolateral directions while maintaining their balance; the maximum value was recorded. The test was repeated three times in each direction and the average value for each direction was calculated and recorded in centimetres. A normalisation formula was used to neutralise the advantage/disadvantage of lower extremity length [35].

$$\begin{array}{c} Normalisation\;formula:Anterior+Posteromedial+Posterolateral)/3\times Lower\;Limb\;Length]\times 100 \end{array}$$

**Application plan:** The Oculus Quest 2 VR games listed below took place over eight weeks during the spring term of the 2024/25 academic year, from 24 March to 20 May 2025. Each game was played for ten minutes per week in physical education and sports classes. When determining the duration of the intervention, consideration was given to the potential for cyber-illness and the limited attention spans that may be exhibited by groups of children and adolescents using virtual reality technologies. Consequently, the literature recommending short sessions was taken as a basis [36,37].

**Fig 3:**
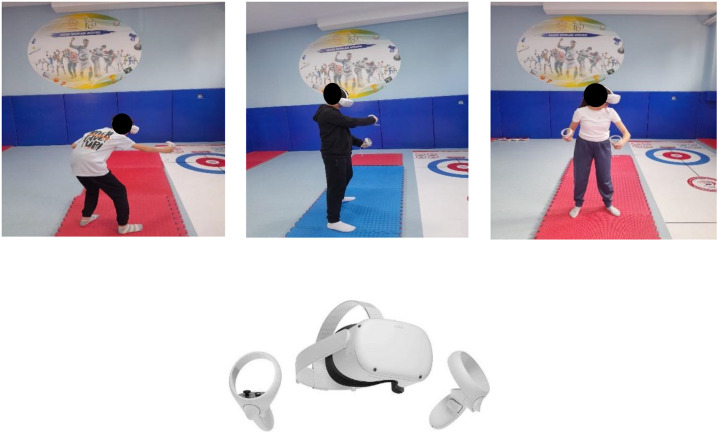
Activity images of students in the experimental group during the VR application. (The researchers took the photographs and obtained verbal consent from the parents.).

- Carve Snowboard: A snowboarding simulator combining various activities involving flexibility, balance, agility, and coordination.

- First Person Tennis – The Real Tennis Simulator: Tennis addresses fundamental motor skills such as speed, balance and coordination. Therefore, this game has been chosen as it is believed to provide all the physical activity that school-age children need. Fig 3 shows examples of the activities that the students in the experimental group performed during the virtual reality sessions.

In this context, studies have been conducted to investigate the impact of virtual reality-based sports simulations on motor skill development. For example, it has been reported that skiing-based VR applications positively impact physical performance and attention [38], while tennis-based VR training has been shown to enhance dynamic balance and motor performance [39]. Taken together, these studies suggest that VR-based sports simulations may be an effective way to develop motor skills.

### Data analysis

The data were analysed using the JAMOVI (version 2.3.21.0) statistical software package. Prior to commencing the analysis, the normality of the variables was assessed using skewness and kurtosis. As both coefficients fell within the range of −1.5 to +1.5, it was concluded that the data exhibited a normal distribution [40]. Descriptive statistics regarding the participants’ demographic characteristics and measurement results were presented using mean (X̄), standard deviation (SD), frequency (n) and percentage (%) values. A two-way mixed-design analysis of variance (ANOVA) was applied to examine changes in balance and coordination performance between the experimental and control groups. The analyses examined the effects of group, time, and group × time interaction. A statistical significance level of p < .05 was adopted.

### Research ethics

Permission to commence the study was obtained from the Scientific Research and Publication Ethics Committee of the Faculty of Health Sciences at Karamanoğlu Mehmetbey University on 02/10/2024 (Document No: E-15466469-100-220190). The necessary official permission to conduct the study was also obtained from the Karaman Provincial Directorate of National Education on 19/11/2024. Students who volunteered to participate were included in the study. The verbal consent process was conducted in an orderly manner and witnessed by the physical education teacher.

### Findings

The average height of the students in the experimental group was 158.6 ± 8.80 cm, while the average height of the control group was 157.6 ± 6.43 cm. No significant difference in height was observed between the two groups. Examining body weight, the average weight of the experimental group was found to be 49.8 ± 13.5 kg, while that of the control group was found to be 58.4 ± 12.2 kg. The control group was significantly heavier than the experimental group. In terms of gender distribution, the experimental group was 60% male and 40% female, while the control group was 36.8% male and 63.2% female. This indicates that the gender distribution is not perfectly balanced between the groups. Examining the body mass index (BMI) values shows that the average BMI of the experimental group is 19.8 kg/m^2^, while that of the control group is 23.5 kg/m^2^. The BMI of the control group is higher than that of the experimental group (see Table 1).

**Table 1:** Baseline demographic characteristics of the experimental and control groups, alongside the results of the between-group comparisons.

Variable	Experimental (n = 20)	Control (n = 19)
Height (cm)	158.6 ± 8.80	157.6 ± 6.43
Weight (kg)	49.8 ± 13.5	58.4 ± 12.2
Female, n (%)	8 (40.0)	12 (63.2)
Male, n (%)	12 (60.0)	7 (36.8)
BMI (kg/m²)*	19.64	23.31

According to Table 2, all variables increased between the pre-test and post-test measurements in the experimental group. The highest increases were seen in the LJ, KTK and Y Balance Test (Right–Left) scores. In the control group, however, the measurements generally remained at a similar level, indicating limited change. These findings suggest that the intervention process is associated with positive changes in performance indicators within the experimental group.

**Table 2:** Descriptive statistics for pre-test and post-test scores of experimental and control groups.

Variable	Experimental Pre-test X̄ ± SS	Experimental Post-test X̄ ± SS	Control Pre-test X̄ ± SS	Control Post-test X̄ ± SS
BB	33.8 ± 15.8	36.1 ± 11.0	20.6 ± 11.9	14.2 ± 11.0
SLH	57.6 ± 5.91	59.9 ± 4.91	43.6 ± 11.7	46.6 ± 11.7
LJ	68.4 ± 9.30	89.5 ± 15.3	63.3 ± 10.5	69.3 ± 18.0
PT	21.1 ± 3.66	24.6 ± 5.12	20.0 ± 2.36	20.5 ± 2.97
KTK	181 ± 22.1	210.0 ± 22.7	147 ± 27.1	150.6 ± 27.4
Y Balance (R)	92.4 ± 12.8	99.7 ± 10.4	89.7 ± 11.7	87.6 ± 12.8
Y Balance (L)	94.7 ± 13.4	103 ± 10.9	91.1 ± 12.1	89.3 ± 12.6

BB: Backward Balance; SLH: Single-Leg Hopping; LJ: Lateral Jumping; PT: Platform Transport; KTK: Körperkoordinationstest für Kinder

The results of the mixed-design ANOVA showed significant interactions between group and time for all variables, except for the single-leg jump variable. Significant interactions were found for Backward Balance (F(1, 37) = 5.74, p = .022, η²_p = .134), Lateral Jumping (F(1, 37) = 17.20, p < .001, η²_p = .317), Platform Transport (F(1, 37) = 8.06, p = .007, η²_p = .179), Total KTK Score (F(1, 37) = 27.40, p < .001, η²_p = .425), Right-Side Balance (F(1, 37) = 6.98, p = .012, η²_p = .159), and Left-Side Balance Performance (F(1, 37) = 8.67, p = .006, η²_p = .190). In contrast, no significant group × time interaction was found for Single-Leg Hopping performance (F(1, 37) = 0.23, p = .634, η²_p = .006). These findings indicate significant differences in changes to balance and coordination performance over time between the experimental and control groups. The experimental group demonstrated greater improvement than the control group on most measurement variables (see Table 3).

**Table 3:** Results of the mixed-design ANOVA for balance and coordination performance in the experimental and control groups.

Variable	Time F(1,37)	p	Group F(1,37)	p	Time × Group F(1,37)	p	η²p
BB	1.18	.285	23.70	<.001	5.74	.022	.134
SLH	9.74	.003	24.40	<.001	0.23	.634	.006
LJ	55.5	<.001	9.90	.003	17.20	<.001	.317
PT	13.89	<.001	5.86	.020	8.06	.007	.179
KTK	42.0	<.001	37.50	<.001	27.40	<.001	.425
Y Balance (R)	2.12	.154	4.78	.035	6.98	.012	.159
Y Balance (L)	3.53	.068	5.82	.021	8.67	.006	.190

BB: Backward Balance; SLH: Single-Leg Hopping; LJ: Lateral Jumping; PT: Platform Transport; KTK: Körperkoordinationstest für Kinder

## Discussion and Conclusions

This study examined the effect of playing the VR game on the development of balance and coordination skills in 7th-grade students. Following an eight-week intervention period, the findings revealed that the experimental group demonstrated greater improvement than the control group in Backward Balancing, Lateral jumping, Platform Transport, total KTK score and right and left Y-balance performance. The significant group × time interactions for these variables in the mixed-design ANOVA results suggest that the improvement observed is not solely dependent on time, but may also be due to the intervention process. A randomised controlled study by Tomaç et al. [41] determined that a 6-week virtual reality–based training programme significantly improved balance and speed-agility in obese children. Following the implementation of a virtual reality–based physical education programme for Years 5 and 6 students, significant differences were observed between the experimental and control groups in terms of cardiovascular endurance, flexibility, muscle strength, endurance, power and BMI [16]. A recent systematic review found that virtual reality applications significantly and effectively improve motor coordination skills in children. This technology supports motor learning processes and contributes to the development of motor performance, particularly by capturing children’s interest [42,43]. VR applications have been observed to improve reaction time, hand-eye coordination [44] and balance performance [25] in students. Studies have shown that eight weeks of VR-based coordination training significantly improves motor skills, particularly hand-eye coordination, reaction time, and shooting accuracy [45]. Novak et al. [39] analysed the effects of a five-minute VR training programme on dynamic balance in tennis players, concluding that such technologies have the potential to enhance physical performance. These results demonstrate that VR technology can be used as an educational tool that supports physical and motor development, as well as for entertainment purposes. A study by Bürger et al. [46] determined that skills acquired in the VR environment could be transferred to the real world following a 6-week training programme consisting of 12 sessions. The study also found that VR training was as effective as real-world training in improving the quality of movements performed on balance equipment. Fernández-Vázquez et al. [15] investigated the impact of integrating VR games and gamification techniques into physical education, revealing that this approach can substantially enhance students’ motor skills and perceived effort. Similarly, a study using augmented reality-based mobile applications reported meaningful improvements in primary school students’ gross motor skills [47]. While the technologies employed vary, both approaches demonstrate that interactive digital applications can support motor skill development. Deng and colleagues [48] emphasised in their studies that digital and competitive virtual reality platforms have the potential to encourage young people to participate in physical activity and boost their self-confidence. Furthermore, Dong et al. [49] observed that VR has the potential not only to develop specific motor skills and cognitive abilities, but also to optimise the trajectory of the targeted movement. In addition to these physical contributions, the effects of VR on cognitive processes are also noteworthy. Haryana et al. [50] stated that VR enables the more efficient use of mental capacity, enhancing both learning and performance in physical exercises. VR technology creates an interactive and immersive learning environment for children, and cognitive game theory and gamified education have been shown to enhance cognitive function and motor coordination in children with cognitive impairments [51]. Thus, VR applications are seen as an important tool for increasing students’ motivation to participate in physical activity and class activities, as well as supporting performance areas related to motor skills.

The findings suggest that playing the VR game can significantly improve students’ balance and coordination, supporting the integration of such technology into the educational process as a means of promoting students’ physical development. However, the literature, particularly systematic reviews conducted since 2020, mostly focuses on VR applications for adults [52–56] or individuals with special needs [57–60]. Application studies targeting healthy child populations are limited [61]. In this context, the present study is important in filling this gap, providing a foundation for further research on VR-based motor skill development in childhood.

### Limitations

Certain limitations were also observed during this study. For example, students were only able to experience the VR game for a limited period of time, once a week. It is thought that longer or more frequent applications could make the development of skills more pronounced. Furthermore, although the user manual for the glasses recommended a minimum age of 13, there were some difficulties in ensuring a perfect fit for the participants’ age and head size. In addition, as the study was conducted during the spring term, the students’ increased desire to spend time outdoors sometimes reduced their motivation and caused distractions, particularly during the application sessions. This situation can be considered an environmental factor that could affect the intensity of the application and the level of participation. In future studies, it is recommended that the results be strengthened by selecting devices that are more suitable for individual differences and increasing the application periods. Another significant limitation of the study is its relatively small sample size. This may have reduced the study’s statistical power and limited the generalisability of its findings. It is anticipated that future studies with larger samples will provide stronger evidence. On the other hand, the random participation of students who are inclined towards sports and volunteers in the experimental group indicates that the significant differences obtained may be related to certain individual characteristics in the selected criteria. This situation could potentially be interpreted as selection bias. In future studies, it is recommended to increase the generalisability of the findings by balancing the physical characteristics of the participants and selecting a more controlled sample.
