# Supplementary material for: Virtual reality experience in physical education and sports lessons: An investigation into its effect on the balance and coordination skills of middle school students
Source: PLoS One. 2026 Aug 12;21(8):e0354258. doi: 10.1371/journal.pone.0354258 (PMC13466030; doi:10.1371/journal.pone.0354258)
Supplement: Supplementary file 1 [file pone.0354258.s002.docx]

**Response to Reviewer #1**

We sincerely thank the reviewer for the constructive comments and valuable suggestions. All comments have been carefully considered and the manuscript has been revised accordingly. Our responses are presented below.

**Abstract**

**1. Comment:** It is unclear whether KTK / Body Coordination Test for Children is a single test or a battery of tests.

**Response:** The abstract has been revised to clarify that the Body Coordination Test for Children (KTK) consists of four subtests and that the total coordination score is derived from the combined performance across these subtests.

**2. Comment:** Backward balance appears to be a component of KTK, but this is not clear in the abstract.

**Response:** The abstract has been revised to clarify that backward balance is one of the KTK subtests.

**Introduction**

**3. Comment:** Active video games were not considered.

**Response:** Additional literature on active video games (exergames) and their effects on physical activity, balance, and motor skills has been added to the Introduction section.

**4. Comment:** Clarify that “it” refers to VR and explain its relationship with balance and coordination.

**Response:** The sentence has been revised to explicitly state that virtual reality technology has been associated with improvements in balance and coordination.

**5. Comment:** The sentence regarding action VR games appears disconnected from the paragraph.

**Response:** Additional explanations have been provided regarding the relevance of action VR games and the contribution of repeated practice to motor and cognitive adaptations.

**Methods**

**6. Comment:** The sentence describing the pre-test/post-test design is unnecessary.

**Response:** The sentence has been removed from the manuscript.

**7. Comment:** Clarify whether participants were in Year 7 or Grade 7.

**Response:** Terminology has been standardized throughout the manuscript. Participants are now consistently described as 7th-grade students.

**8. Comment:** Clarify the exclusion criteria regarding a history of dizziness.

**Response:** The exclusion criteria have been clarified in the revised manuscript.

**Data Collection Tools**

**9. Comment:** Clarify KTK scoring and naming consistency.

**Response:** Additional information has been provided explaining that each KTK subtest is scored separately and that the scores are combined to obtain the total KTK score. Terminology has been standardized throughout the manuscript.

**Figure 1**

**11. Comment:** Verify the ICC values reported for the Y Balance Test.

**Response:** The reliability values and the corresponding reference have been rechecked and corrected where necessary.

**Application Plan**

**12. Comment:** Justify the 10-minute VR intervention duration.

**Response:** A justification based on VR safety considerations, usability recommendations, and previous studies has been added to the manuscript.

**Games**

**13. Comment:** Provide references supporting the use of snowboard and tennis VR applications.

**Response:** Relevant literature regarding VR ski/snowboard simulations and VR tennis-based training applications has been added.

**Data Analysis**

**14. Comment:** A 2 × 2 mixed-design ANOVA should be used.

**Response:** Following the reviewer’s recommendation, the statistical analyses were revised. A 2 (group) × 2 (time) mixed-design ANOVA was conducted to examine the effects of the intervention.

**Table 1**

**15. Comment:** Clarify whether KTK represents a total score.

**Response:** The table description has been revised to clarify that the KTK score represents the total score derived from the four subtests.

**Table 2**

**16. Comment:** Clarify balance test terminology and capitalization consistency.

**Response:** Terminology and capitalization have been standardized throughout the manuscript.

**17. Comment:** Clarify the statement regarding recalculation.

**Response:** The unclear statement has been removed and the Results section has been revised for greater clarity.

**18. Comment:** Include the relevant table reference.

**Response:** Appropriate table references have been added to the manuscript.

**19. Comment:** Clarify the grade-level inconsistency.

**Response:** All references have been corrected, and participants are consistently described as 7th-grade students.

**Discussion**

**20. Comment:** The discussion resembles a list of references and does not sufficiently relate the findings to the literature.

**Response:** The Discussion section has been substantially revised. The findings are now directly interpreted in relation to relevant previous studies, and the potential implications of the results are discussed in greater detail.

We sincerely thank the reviewer for these valuable comments and suggestions. Their feedback has significantly contributed to improving the quality and clarity of the manuscript.

**Response to Reviewer #2**

Dear Reviewer,

We sincerely thank you for your careful evaluation of our manuscript and for your detailed and constructive feedback. Your comments have significantly contributed to improving the methodological quality and scientific presentation of our study. All suggestions were carefully considered, and the manuscript has been revised accordingly. Our responses are presented below.

**1. Abstract**

**Comment:** The abstract is overly descriptive and lengthy. The study design, hypothesis, and participant characteristics are not sufficiently clear. The results strongly emphasize the effectiveness of VR despite baseline differences between groups.

**Response:** The abstract has been revised in accordance with the journal’s guidelines. Unnecessary details and interpretive statements have been removed. The study design, participant characteristics, objectives, and primary hypothesis are now presented more clearly. A more cautious interpretation of the findings has been adopted, and the baseline differences between groups have been acknowledged as an important consideration when interpreting the results.

**2. Introduction**

**Comment:** The Introduction lacks a clear structure, does not adequately justify the research gap, and does not clearly present the study hypotheses.

**Response:** The Introduction has been extensively revised and restructured. The theoretical foundation of the study has been strengthened, the research gap has been more clearly defined, and the originality of the study has been emphasized. In addition to the limited evidence available in Türkiye, broader research gaps related to VR-based motor skill development in children have been highlighted. Explicit study hypotheses have been added. Furthermore, the literature review has been revised to provide a more critical synthesis of directly relevant studies.

**3. Methods**

**Comment:** There are important methodological concerns regarding sample size, sampling procedures, baseline group differences, participant characteristics, intervention protocol, and measurement tools.

**Response:** The Methods section has been thoroughly reviewed and revised.

- Participants’ age, sex, and other key demographic characteristics have been reported in greater detail.
- The sampling and group allocation procedures have been clarified.
- Baseline differences observed between groups have been explicitly reported in the Results and Discussion sections and considered when interpreting the findings.
- The intervention protocol has been described in greater detail, including intervention duration, content, VR games used, and implementation procedures.
- It has been clarified that all participants followed the same intervention stages.
- Descriptions of the measurement tools have been reviewed, and the typographical error regarding the ICC values of the Y Balance Test has been corrected.
- The normalization formula has been rechecked and presented correctly.
- The Ethics section now clearly states that parental informed consent was obtained.
- Additional details regarding the intervention procedures have been included to improve study replicability.

**4. Statistical Analysis**

**Comment:** The statistical methods used may not be optimal given the baseline differences between groups.

**Response:** Following the reviewer’s recommendation, the statistical analyses were reconsidered. A 2 (group) × 2 (time) mixed-design ANOVA was conducted to examine the intervention effects, allowing a more appropriate evaluation of time, group, and interaction effects. In addition, the interpretation of effect sizes has been reviewed and presented more cautiously. The Statistical Analysis section has also been expanded and clarified.

**5. Results**

**Comment:** The Results section is overly descriptive, and some interpretations do not adequately account for baseline differences.

**Response:** The Results section has been revised. Redundant explanations have been reduced, and unnecessary repetition between tables and text has been removed. Baseline differences have been taken into account when interpreting the findings, and statements regarding intervention effectiveness have been rewritten more cautiously. All statistical results were rechecked, and necessary corrections were made.

**6. Discussion**

**Comment:** The Discussion is primarily confirmatory and does not adequately consider alternative explanations.

**Response:** The Discussion section has been extensively revised. Findings are now compared not only with supporting studies but also with studies reporting different or conflicting results. The potential influence of baseline group differences on the findings has been discussed. Alternative explanations, including maturation effects, learning effects, and other possible factors, have also been considered. Furthermore, the implications of the methodological and statistical limitations have been discussed in greater detail.

**7. Limitations**

**Comment:** The limitations section is incomplete.

**Response:** The Limitations section has been expanded. The small sample size, potential selection bias, baseline group differences, limited intervention duration, and statistical limitations have been explicitly acknowledged. Recommendations for future research have also been provided.

We sincerely appreciate the reviewer’s insightful comments and constructive suggestions. We believe that the revisions made in response to these comments have substantially improved the scientific quality, methodological transparency, and overall clarity of the manuscript.
