# Supplementary material for: Virtual reality experience in physical education and sports lessons: An investigation into its effect on the balance and coordination skills of middle school students
Source: PLoS One. 2026 Aug 12;21(8):e0354258. doi: 10.1371/journal.pone.0354258 (PMC13466030; doi:10.1371/journal.pone.0354258)
Supplement: Peer review history

# Peer review: Decision Letter 0

**DOI:** 10.1371/journal.pone.0354258.r001

**Authors:** Francesca D’Elia

20 Apr 2026

PONE-D-25-56146

Virtual reality experience in physical education and sports lessons: An investigation into its effect on the balance and coordination skills of middle school students

PLOS One

Dear Dr. YANAR,

Thank you for submitting your manuscript to PLOS ONE. After careful consideration, we feel that it has merit but does not fully meet PLOS ONE’s publication criteria as it currently stands. Therefore, we invite you to submit a revised version of the manuscript that addresses the points raised during the review process.

Please submit your revised manuscript by Jun 04 2026 11:59PM. If you will need more time than this to complete your revisions, please reply to this message or contact the journal office at plosone@plos.org. When you're ready to submit your revision, log on to https://www.editorialmanager.com/pone/ and select the 'Submissions Needing Revision' folder to locate your manuscript file.

Please include the following items when submitting your revised manuscript:

- A letter that responds to each point raised by the academic editor and reviewer(s). You should upload this letter as a separate file labeled 'Response to Reviewers'.
- A marked-up copy of your manuscript that highlights changes made to the original version. You should upload this as a separate file labeled 'Revised Manuscript with Track Changes'.
- An unmarked version of your revised paper without tracked changes. You should upload this as a separate file labeled 'Manuscript'.

If you would like to make changes to your financial disclosure, please include your updated statement in your cover letter. Guidelines for resubmitting your figure files are available below the reviewer comments at the end of this letter.

If applicable, we recommend that you deposit your laboratory protocols in protocols.io to enhance the reproducibility of your results. Protocols.io assigns your protocol its own identifier (DOI) so that it can be cited independently in the future. For instructions see: https://journals.plos.org/plosone/s/submission-guidelines#loc-laboratory-protocols. Additionally, PLOS ONE offers an option for publishing peer-reviewed Lab Protocol articles, which describe protocols hosted on protocols.io. Read more information on sharing protocols at https://plos.org/protocols?utm_medium=editorial-email&utm_source=authorletters&utm_campaign=protocols.

As the corresponding author, your ORCID iD is verified in the submission system and will appear in the published article. PLOS supports the use of ORCID, and we encourage all coauthors to register for an ORCID iD and use it as well. Please encourage your coauthors to verify their ORCID iD within the submission system before final acceptance, as unverified ORCID iDs will not appear in the published article. *Only*  the individual author can complete the verification step; PLOS staff *cannot*  verify ORCID iDs on behalf of authors.

We look forward to receiving your revised manuscript.

Kind regards,

Francesca D'Elia, Ph.D.

Academic Editor

PLOS One

Journal Requirements:

When submitting your revision, we need you to address these additional requirements.

1. Please ensure that your manuscript meets PLOS ONE's style requirements, including those for file naming. The PLOS ONE style templates can be found at

https://journals.plos.org/plosone/s/file?id=wjVg/PLOSOne_formatting_sample_main_body.pdf and

https://journals.plos.org/plosone/s/file?id=ba62/PLOSOne_formatting_sample_title_authors_affiliations.pdf

2. Thank you for stating the following in the Acknowledgments Section of your manuscript:

“This research was supported by project number 08-M-24 and was approved by the Scientific Research Projects Commission at Karamanoğlu Mehmetbey University.”

We note that you have provided funding information that is not currently declared in your Funding Statement. However, funding information should not appear in the Acknowledgments section or other areas of your manuscript. We will only publish funding information present in the Funding Statement section of the online submission form.

Please remove any funding-related text from the manuscript and let us know how you would like to update your Funding Statement. Currently, your Funding Statement reads as follows:

“The author(s) received no specific funding for this work.”

Please include your amended statements within your cover letter; we will change the online submission form on your behalf.

3. Thank you for stating the following financial disclosure:

“This research was supported by project number 08-M-24 and was approved by the Scientific Research Projects Commission at Karamanoğlu Mehmetbey University.”

At this time, please address the following queries:

a) Please clarify the sources of funding (financial or material support) for your study. List the grants or organizations that supported your study, including funding received from your institution.

b) State what role the funders took in the study. If the funders had no role in your study, please state: “The funders had no role in study design, data collection and analysis, decision to publish, or preparation of the manuscript.”

c) If any authors received a salary from any of your funders, please state which authors and which funders.

d) If you did not receive any funding for this study, please state: “The authors received no specific funding for this work.”

Please include your amended statements within your cover letter; we will change the online submission form on your behalf.

4. In the online submission form you indicate that your data is not available for proprietary reasons and have provided a contact point for accessing this data. Please note that your current contact point is a co-author on this manuscript. According to our Data Policy, the contact point must not be an author on the manuscript and must be an institutional contact, ideally not an individual. Please revise your data statement to a non-author institutional point of contact, such as a data access or ethics committee, and send this to us via return email. Please also include contact information for the third party organization, and please include the full citation of where the data can be found.

5. Please amend the manuscript submission data (via Edit Submission) to include author Nazlı YANAR TUNÇEL.

6. Please amend your authorship list in your manuscript file to include author Nazlı YANAR.

7. Please ensure that you refer to Figure 1 in your text as, if accepted, production will need this reference to link the reader to the figure.

8. We note that Shape 2 in your submission contain copyrighted images. All PLOS content is published under the Creative Commons Attribution License (CC BY 4.0), which means that the manuscript, images, and Supporting Information files will be freely available online, and any third party is permitted to access, download, copy, distribute, and use these materials in any way, even commercially, with proper attribution. For more information, see our copyright guidelines: http://journals.plos.org/plosone/s/licenses-and-copyright.

We require you to either (1) present written permission from the copyright holder to publish these figures specifically under the CC BY 4.0 license, or (2) remove the figures from your submission:

1. You may seek permission from the original copyright holder of Figure(s) [#] to publish the content specifically under the CC BY 4.0 license.

We recommend that you contact the original copyright holder with the Content Permission Form (http://journals.plos.org/plosone/s/file?id=7c09/content-permission-form.pdf) and the following text:

“I request permission for the open-access journal PLOS ONE to publish XXX under the Creative Commons Attribution License (CCAL) CC BY 4.0 (http://creativecommons.org/licenses/by/4.0/). Please be aware that this license allows unrestricted use and distribution, even commercially, by third parties. Please reply and provide explicit written permission to publish XXX under a CC BY license and complete the attached form.”

Please upload the completed Content Permission Form or other proof of granted permissions as an "Other" file with your submission.

In the figure caption of the copyrighted figure, please include the following text: “Reprinted from [ref] under a CC BY license, with permission from [name of publisher], original copyright [original copyright year].”

2. If you are unable to obtain permission from the original copyright holder to publish these figures under the CC BY 4.0 license or if the copyright holder’s requirements are incompatible with the CC BY 4.0 license, please either i) remove the figure or ii) supply a replacement figure that complies with the CC BY 4.0 license. Please check copyright information on all replacement figures and update the figure caption with source information. If applicable, please specify in the figure caption text when a figure is similar but not identical to the original image and is therefore for illustrative purposes only.

9. We note that Shape 1 and 3 includes an image of a participant in the study.

As per the PLOS ONE policy (http://journals.plos.org/plosone/s/submission-guidelines#loc-human-subjects-research) on papers that include identifying, or potentially identifying, information, the individual(s) or parent(s)/guardian(s) must be informed of the terms of the PLOS open-access (CC-BY) license and provide specific permission for publication of these details under the terms of this license. Please download the Consent Form for Publication in a PLOS Journal (http://journals.plos.org/plosone/s/file?id=8ce6/plos-consent-form-english.pdf). The signed consent form should not be submitted with the manuscript, but should be securely filed in the individual's case notes. Please amend the methods section and ethics statement of the manuscript to explicitly state that the patient/participant has provided consent for publication: “The individual in this manuscript has given written informed consent (as outlined in PLOS consent form) to publish these case details”.

If you are unable to obtain consent from the subject of the photograph, you will need to remove the figure and any other textual identifying information or case descriptions for this individual.

10. If the reviewer comments include a recommendation to cite specific previously published works, please review and evaluate these publications to determine whether they are relevant and should be cited. There is no requirement to cite these works unless the editor has indicated otherwise.

Additional Editor Comments:

Thank you for submitting your manuscript.

The topic is timely and of clear interest, and both reviewers recognize the potential value of your work. At the same time, their comments highlight several areas where the manuscript requires substantial clarification and improvement. In particular, the study design, methodological details, and statistical analyses need to be presented more clearly and rigorously, and the narrative structure, especially in the introduction and discussion, would benefit from greater focus and coherence.

I encourage you to revise the manuscript thoroughly, addressing all reviewer comments in detail and ensuring that the revised version provides a clearer, more robust, and more transparent account of your study. We look forward to considering a substantially improved version.

[Note: HTML markup is below. Please do not edit.]

Reviewers' comments:

Reviewer's Responses to Questions

**Comments to the Author**

1. Is the manuscript technically sound, and do the data support the conclusions?

The manuscript must describe a technically sound piece of scientific research with data that supports the conclusions. Experiments must have been conducted rigorously, with appropriate controls, replication, and sample sizes. The conclusions must be drawn appropriately based on the data presented.

Reviewer #1: Yes

Reviewer #2: Partly

**********

2. Has the statistical analysis been performed appropriately and rigorously?

Reviewer #1: No

Reviewer #2: No

**********

3. Have the authors made all data underlying the findings in their manuscript fully available?

The PLOS Data policy requires authors to make all data underlying the findings described in their manuscript fully available without restriction, with rare exception (please refer to the Data Availability Statement in the manuscript PDF file). The data should be provided as part of the manuscript or its supporting information, or deposited to a public repository. For example, in addition to summary statistics, the data points behind means, medians and variance measures should be available. If there are restrictions on publicly sharing data—e.g. participant privacy or use of data from a third party—those must be specified.

Reviewer #1: Yes

Reviewer #2: Yes

**********

4. Is the manuscript presented in an intelligible fashion and written in standard English?

PLOS ONE does not copyedit accepted manuscripts, so the language in submitted articles must be clear, correct, and unambiguous. Any typographical or grammatical errors should be corrected at revision, so please note any specific errors here.

Reviewer #1: Yes

Reviewer #2: Yes

**********

5. Review Comments to the Author

Please use the space provided to explain your answers to the questions above. You may also include additional comments for the author, including concerns about dual publication, research ethics, or publication ethics. (Please upload your review as an attachment if it exceeds 20,000 characters)

Reviewer #1: Overall, I think adding to the literature on VR and activities is a great idea. Knowing what VR can do from a balance and motor aspect will help many populations and possibly even work towards a run at reducing obesity.

This article topic is great but there is significant improvements needed in many areas.

Sections

Abstract

In the abstract there is a couple things that need clarification:

When describing the KTK / children’s body coordination test it is not clear if it is one total test or a series of tests. (it does talk about it some later but still should be semi clear to reader in abstract)

Also here it talks about the backward balance test. I think that is a part of the KTK from reading later in the article but in abstract it is confusing since the y balance test is mentioned but not a backward balance test. Please correct this in the writing.

INTRO

Have you looked into or compared active video gaming? Line 57 talks about regular games compared to VR but no mention of active gaming. That shouldn’t be left out as it is part of video games and does show balance and Physical activity improvements.

Line 69: clarify that “it” means VR and that VR improves balance and coordination.

Line 72: this sentence and reference seems out of place like it was just thrown in to the paragraph. There is an interesting fact in regards to that maybe expand upon its importance.

METHODS

Line 85: this sentence is not needed as it is really common knowledge that pre post test is a common research method.

Line 89: please clarify year 7. Is that 6th grade or 7th grade. Also later in the document you call it 8th year. Please be consistent on the numbers and definitions

Exclusion criteria is having a history of vertigo not an exclusion criteria. And if not why not?

Data collection tools

The use of the KTK is great. But I am not clear as to the scoring. Is each part its own score and then is there a total score for the KTK? If there is a total score, please explain that to the reader. Also with this is the parts of the test to be capitalized or not. Inconsistencies are occurring in the document. Please correct throughout the entire text.

SHAPE 1

This figure is not at all clear. Please clarify what y axis is and what is this trying to tell me. Is it a frequency chart? What does the lines mean and what do the numbers mean?

Line 106 should that be .85 to 1.0 versus .85 to -.01 not consistent with the .99 to 1.0 for the inter rater range.

Application plan

What was the rationale behind only 10 minutes of game play? Is there a reference that says 10 min only or is that more in line with what time was allotted in the classroom session?

Games

Any references of past studies using Snowboard or tennis in the literature?

Data analysis

The most correct way to report and analysis this data should be with a 2 (condition) x 2 (time) mixed model ANOVA. And then running T test for post analysis

With the data charts and writing. Please correct the redundancy of having all the stats in the charts and the writing.

TABLE 1. you have the pre and posttest KTK but I want to see clarification is that is a total score or sum of all the other 4 scores or some other individual test result.

TABLE 2. is it balance or denge test in the chart?

Line 19: should those words be capitalized? If so correct throughout the document.

Line 216: I am unsure what you mean by warranting a revaluation of the calculation. Do you mean you need to redo the stats or are you thinking you need to reevaluate the use of a particular calculation.

Line 225 please reference table 5 here. You do a reference to each other table but not here. Please be consistent. The reference at the end of the paragraph is confusing.

Line 237. Referring to year 8 here is it 7 or 8.

Discussion

Overall, the discussion seems to just be a listing of other references it doesn’t tie any of your data or findings to current literature. Could you please bring together your results and findings to compare to the specific references you use in your discussion.

Your last paragraph starts to bring together the info learned from your study, but I would suggest tying it in also in the previous paragraphs of the discussion.

Reviewer #2: Dear authors,

The topic appears to be a current and relevant one (the use of virtual reality (VR) in physical education for the development of motor skills in children), the subject falling within important interdisciplinary fields (physical education, educational technology, motor development). We determined that an experimental design with control group and pre-test/post-test is used, with a limited intervention duration (8 weeks), with two standardized instruments (Körperkoordinationstest für Kinder, a valid instrument for coordination and Y Balance Test, a valid test for dynamic balance). We also determined that the authors report statistical effects for t-test, p-value and Cohen’s d (effect size) and include limitations of the study, which is methodologically correct.

However, I ask authors to pay attention to the following comments and observations:

1) The abstract is too descriptive and too long for journal standards, contains premature interpretations (“practically significant improvements in all criteria”), includes excessive details about statistical results, but does not clearly summarize the design and hypothesis, and does not clearly mention the characteristics of the participants. It is stated that the results clearly demonstrate the effectiveness of VR, but the pre-test shows initial differences between groups, which weakens this claim.

2) The introduction indicates incomplete aspects, weak for the structure, i.e. many general statements about technology in education, but without explicit hypotheses, and the justification of the scientific gap is poorly formulated (the statement: “insufficiently researched, particularly in Turkish sources” and a geographical gap is not sufficient for scientific justification). The literature is cited descriptively, not critically, and some references are poorly connected to the objective (e.g. studies on cognitive or general VR).

3) Methods is the section with the most important methodological problems. We determined that the sample is small, the randomization is ambiguous (it states “random sampling” but the description indicates convenience sampling), demographic characteristics (sex, age, physical activity level, BMI) are missing, the groups are not balanced at baseline, significant differences in the pre-test for backward balance, single-leg hop, total KTK. I believe that all of these aspects present a major internal validity problem and also affect replicability.

From the point of view of the intervention, it is extremely short (10 minutes per week for 8 weeks!!!!), the protocol is insufficiently described (missing: activity intensity, exact number of repetitions, effort monitoring) and it is not clear whether all people played the same levels and whether previous VR experience was controlled.

The instruments are not fully reported, the description of the Y Balance test contains an error in the ICC (“0.85–0.01” - logically impossible interval), and the normalization formula is written incorrectly.

I determined that verbal consent is mentioned, but for minors, written parental consent should be provided, from the point of view of research ethics!!!

Problems for replicability, because they are not completely described: participant characteristics, randomization procedure, control of external variables, exact protocol of the intervention, intensity monitoring.

I ask the authors to clarify, provide details and check the entire Methods section once again. Thank you

4) The statistical analysis indicates problems, in the sense that the independent t-test for comparing post-tests in the context of initial differences between groups is not clear (maybe the authors could determine ANCOVA or mixed ANOVA), there is no analysis of statistical power (power analysis), there are no corrections for multiple testing, and some interpretations of the effect size are exaggerated.

The relevance of the statistical analyses is partially adequate, because the t-test is acceptable for pre/post comparison in the same group, but for the experimental design it is suboptimal. Mixed ANOVA or ANCOVA with pretest as covariate would have been correct. The problem becomes major because there are significant initial differences.

5) Results are described redundantly (text + table), some interpretations are incorrect (the effectiveness of the intervention is stated despite the existence of baseline imbalance), and some results are contradictory (experimental backward balance is not significant in paired t-test). og authors to clarify, provide details and double-check the entire Results section. Thanks

6) Discussion is predominantly confirmatory, the initial differences between groups are not critically discussed, alternative explanations (maturation, learning effect) are missing and there are no detailed comparisons with contradictory results from the literature. The discussion is more enumerative than analytical, statistical limitations are not discussed and explanations for the effects are missing.

7) Limitations are mentioned, but they are not complete. Missing: selection bias, baseline imbalance, acceptance of a small sample and statistical problems.

8) Exaggerations in Discussion and Conclusions. Example: "VR game significantly improved the students' balance and coordination"!! The initial differences between the groups may partly explain the results, so the conclusion is stronger than the data allow. The conclusions are partly exaggerated because the design and statistical analysis do not support firm claims of causality.

9) I ask the authors to analyze the relevance of all references and the age of some of them (about 25% are older than 10 years, especially since there are many recent studies and research on this topic). I recommend careful analysis of:

https://doi.org/10.3390/app15179826

https://doi.org/10.1186/s12887-025-05897-x

https://doi.org/10.3390/children12101405

https://doi.org/10.3390/children11070863

References 7,8, 24, 30 are potentially irrelevant, because they are weakly connected to the topic. They are not wrong, but they do not directly contribute to the main argument. I ask the authors to check this aspect once again. Thank you.

10) Tables do not have complete legends (they are minimalist) and abbreviations are not fully defined.

11) Figures are more illustrative than scientific, they have no analytical role.

I think the article is promising, especially the central idea of using VR in physical education, but the authors need to present a clearer, more detailed, better argued version with a more solid statistical analysis.

Otherwise, a good article.

**********

6. PLOS authors have the option to publish the peer review history of their article (what does this mean?). If published, this will include your full peer review and any attached files.

If you choose “no”, your identity will remain anonymous but your review may still be made public.

**Do you want your identity to be public for this peer review?**  For information about this choice, including consent withdrawal, please see our Privacy Policy.

Reviewer #1: No

Reviewer #2: **Yes:**  Dan Iulian ALEXE

**********

[NOTE: If reviewer comments were submitted as an attachment file, they will be attached to this email and accessible via the submission site. Please log into your account, locate the manuscript record, and check for the action link "View Attachments". If this link does not appear, there are no attachment files.]

To ensure your figures meet our technical requirements, please review our figure guidelines: https://journals.plos.org/plosone/s/figures

You may also use PLOS’s free figure tool, NAAS, to help you prepare publication quality figures: https://journals.plos.org/plosone/s/figures#loc-tools-for-figure-preparation.

NAAS will assess whether your figures meet our technical requirements by comparing each figure against our figure specifications.

# Author comment: Author response to Decision Letter 1

**DOI:** 10.1371/journal.pone.0354258.r002

26 Jun 2026

Response to Reviewer #1

We sincerely thank the reviewer for the constructive comments and valuable suggestions. All comments have been carefully considered and the manuscript has been revised accordingly. Our responses are presented below.

Abstract

1. Comment: It is unclear whether KTK / Body Coordination Test for Children is a single test or a battery of tests.

Response: The abstract has been revised to clarify that the Body Coordination Test for Children (KTK) consists of four subtests and that the total coordination score is derived from the combined performance across these subtests.

2. Comment: Backward balance appears to be a component of KTK, but this is not clear in the abstract.

Response: The abstract has been revised to clarify that backward balance is one of the KTK subtests.

Introduction

3. Comment: Active video games were not considered.

Response: Additional literature on active video games (exergames) and their effects on physical activity, balance, and motor skills has been added to the Introduction section.

4. Comment: Clarify that “it” refers to VR and explain its relationship with balance and coordination.

Response: The sentence has been revised to explicitly state that virtual reality technology has been associated with improvements in balance and coordination.

5. Comment: The sentence regarding action VR games appears disconnected from the paragraph.

Response: Additional explanations have been provided regarding the relevance of action VR games and the contribution of repeated practice to motor and cognitive adaptations.

Methods

6. Comment: The sentence describing the pre-test/post-test design is unnecessary.

Response: The sentence has been removed from the manuscript.

7. Comment: Clarify whether participants were in Year 7 or Grade 7.

Response: Terminology has been standardized throughout the manuscript. Participants are now consistently described as 7th-grade students.

8. Comment: Clarify the exclusion criteria regarding a history of dizziness.

Response: The exclusion criteria have been clarified in the revised manuscript.

Data Collection Tools

9. Comment: Clarify KTK scoring and naming consistency.

Response: Additional information has been provided explaining that each KTK subtest is scored separately and that the scores are combined to obtain the total KTK score. Terminology has been standardized throughout the manuscript.

Figure 1

11. Comment: Verify the ICC values reported for the Y Balance Test.

Response: The reliability values and the corresponding reference have been rechecked and corrected where necessary.

Application Plan

12. Comment: Justify the 10-minute VR intervention duration.

Response: A justification based on VR safety considerations, usability recommendations, and previous studies has been added to the manuscript.

Games

13. Comment: Provide references supporting the use of snowboard and tennis VR applications.

Response: Relevant literature regarding VR ski/snowboard simulations and VR tennis-based training applications has been added.

Data Analysis

14. Comment: A 2 × 2 mixed-design ANOVA should be used.

Response: Following the reviewer’s recommendation, the statistical analyses were revised. A 2 (group) × 2 (time) mixed-design ANOVA was conducted to examine the effects of the intervention.

Table 1

15. Comment: Clarify whether KTK represents a total score.

Response: The table description has been revised to clarify that the KTK score represents the total score derived from the four subtests.

Table 2

16. Comment: Clarify balance test terminology and capitalization consistency.

Response: Terminology and capitalization have been standardized throughout the manuscript.

17. Comment: Clarify the statement regarding recalculation.

Response: The unclear statement has been removed and the Results section has been revised for greater clarity.

18. Comment: Include the relevant table reference.

Response: Appropriate table references have been added to the manuscript.

19. Comment: Clarify the grade-level inconsistency.

Response: All references have been corrected, and participants are consistently described as 7th-grade students.

Discussion

20. Comment: The discussion resembles a list of references and does not sufficiently relate the findings to the literature.

Response: The Discussion section has been substantially revised. The findings are now directly interpreted in relation to relevant previous studies, and the potential implications of the results are discussed in greater detail.

We sincerely thank the reviewer for these valuable comments and suggestions. Their feedback has significantly contributed to improving the quality and clarity of the manuscript.

Response to Reviewer #2

Dear Reviewer,

We sincerely thank you for your careful evaluation of our manuscript and for your detailed and constructive feedback. Your comments have significantly contributed to improving the methodological quality and scientific presentation of our study. All suggestions were carefully considered, and the manuscript has been revised accordingly. Our responses are presented below.

1. Abstract

Comment: The abstract is overly descriptive and lengthy. The study design, hypothesis, and participant characteristics are not sufficiently clear. The results strongly emphasize the effectiveness of VR despite baseline differences between groups.

Response: The abstract has been revised in accordance with the journal’s guidelines. Unnecessary details and interpretive statements have been removed. The study design, participant characteristics, objectives, and primary hypothesis are now presented more clearly. A more cautious interpretation of the findings has been adopted, and the baseline differences between groups have been acknowledged as an important consideration when interpreting the results.

2. Introduction

Comment: The Introduction lacks a clear structure, does not adequately justify the research gap, and does not clearly present the study hypotheses.

Response: The Introduction has been extensively revised and restructured. The theoretical foundation of the study has been strengthened, the research gap has been more clearly defined, and the originality of the study has been emphasized. In addition to the limited evidence available in Türkiye, broader research gaps related to VR-based motor skill development in children have been highlighted. Explicit study hypotheses have been added. Furthermore, the literature review has been revised to provide a more critical synthesis of directly relevant studies.

3. Methods

Comment: There are important methodological concerns regarding sample size, sampling procedures, baseline group differences, participant characteristics, intervention protocol, and measurement tools.

Response: The Methods section has been thoroughly reviewed and revised.

• Participants’ age, sex, and other key demographic characteristics have been reported in greater detail.

• The sampling and group allocation procedures have been clarified.

• Baseline differences observed between groups have been explicitly reported in the Results and Discussion sections and considered when interpreting the findings.

• The intervention protocol has been described in greater detail, including intervention duration, content, VR games used, and implementation procedures.

• It has been clarified that all participants followed the same intervention stages.

• Descriptions of the measurement tools have been reviewed, and the typographical error regarding the ICC values of the Y Balance Test has been corrected.

• The normalization formula has been rechecked and presented correctly.

• The Ethics section now clearly states that parental informed consent was obtained.

• Additional details regarding the intervention procedures have been included to improve study replicability.

4. Statistical Analysis

Comment: The statistical methods used may not be optimal given the baseline differences between groups.

Response: Following the reviewer’s recommendation, the statistical analyses were reconsidered. A 2 (group) × 2 (time) mixed-design ANOVA was conducted to examine the intervention effects, allowing a more appropriate evaluation of time, group, and interaction effects. In addition, the interpretation of effect sizes has been reviewed and presented more cautiously. The Statistical Analysis section has also been expanded and clarified.

5. Results

Comment: The Results section is overly descriptive, and some interpretations do not adequately account for baseline differences.

Response: The Results section has been revised. Redundant explanations have been reduced, and unnecessary repetition between tables and text has been removed. Baseline differences have been taken into account when interpreting the findings, and statements regarding intervention effectiveness have been rewritten more cautiously. All statistical results were rechecked, and necessary corrections were made.

6. Discussion

Comment: The Discussion is primarily confirmatory and does not adequately consider alternative explanations.

Response: The Discussion section has been extensively revised. Findings are now compared not only with supporting studies but also with studies reporting different or conflicting results. The potential influence of baseline group differences on the findings has been discussed. Alternative explanations, including maturation effects, learning effects, and other possible factors, have also been considered. Furthermore, the implications of the methodological and statistical limitations have been discussed in greater detail.

7. Limitations

Comment: The limitations section is incomplete.

Response: The Limitations section has been expanded. The small sample size, potential selection bias, baseline group differences, limited intervention duration, and statistical limitations have been explicitly acknowledged. Recommendations for future research have also been provided.

We sincerely appreciate the reviewer’s insightful comments and constructive suggestions. We believe that the revisions made in response to these comments have substantially improved the scientific quality, methodological transparency, and overall clarity of the manuscript.

Attachment Submitted filename: Response to Reviewers-pone31.05.26.docx

# Editor report: Decision Letter 1

**DOI:** 10.1371/journal.pone.0354258.r003

**Authors:** Francesca D'Elia

6 Jul 2026

Virtual reality experience in physical education and sports lessons: An investigation into its effect on the balance and coordination skills of middle school students

PONE-D-25-56146R1

Dear Dr. YANAR TUNÇEL,

We’re pleased to inform you that your manuscript has been judged scientifically suitable for publication and will be formally accepted for publication once it meets all outstanding technical requirements.

Within one week, you’ll receive an e-mail detailing the required amendments. When these have been addressed, you’ll receive a formal acceptance letter and your manuscript will be scheduled for publication.

An invoice will be generated when your article is formally accepted. Please note, if your institution has a publishing partnership with PLOS and your article meets the relevant criteria, all or part of your publication costs will be covered. Please make sure your user information is up-to-date by logging into Editorial Manager at Editorial Manager® and clicking the ‘Update My Information' link at the top of the page. For questions related to billing, please contact billing support.

If your institution or institutions have a press office, please notify them about your upcoming paper to help maximize its impact. If they’ll be preparing press materials, please inform our press team as soon as possible -- no later than 48 hours after receiving the formal acceptance. Your manuscript will remain under strict press embargo until 2 pm Eastern Time on the date of publication. For more information, please contact onepress@plos.org.

Kind regards,

Francesca D'Elia, Ph.D.

Academic Editor

PLOS One

Additional Editor Comments (optional):

Thank you for submitting the revised version of your manuscript. I have carefully reviewed your responses and the updated manuscript. You have addressed all reviewer comments comprehensively, and the revisions have substantially strengthened the clarity, methodological transparency, and overall quality of the work. As the changes fully resolve the concerns raised in the previous round, no further reviewer input is required.

Your manuscript is now suitable for publication in PLOS One.

Reviewers' comments:

# Editor report: Acceptance letter

**DOI:** 10.1371/journal.pone.0354258.r004

**Authors:** Francesca D'Elia

PONE-D-25-56146R1

PLOS One

Dear Dr. Yanar Tunçel,

I'm pleased to inform you that your manuscript has been deemed suitable for publication in PLOS One. Congratulations! Your manuscript is now being handed over to our production team.

At this stage, our production department will prepare your paper for publication. This includes ensuring the following:

* All references, tables, and figures are properly cited

* All relevant supporting information is included in the manuscript submission,

* There are no issues that prevent the paper from being properly typeset

You will receive further instructions from the production team, including instructions on how to review your proof when it is ready. Please keep in mind that we are working through a large volume of accepted articles, so please give us a few days to review your paper and let you know the next and final steps.

Lastly, if your institution or institutions have a press office, please let them know about your upcoming paper now to help maximize its impact. If they'll be preparing press materials, please inform our press team within the next 48 hours. Your manuscript will remain under strict press embargo until 2 pm Eastern Time on the date of publication. For more information, please contact onepress@plos.org.

You will receive an invoice from PLOS for your publication fee after your manuscript has reached the completed accept phase. If you receive an email requesting payment before acceptance or for any other service, this may be a phishing scheme. Learn how to identify phishing emails and protect your accounts at https://explore.plos.org/phishing.

If we can help with anything else, please email us at customercare@plos.org.

Thank you for submitting your work to PLOS ONE and supporting open access.

Kind regards,

PLOS ONE Editorial Office Staff

on behalf of

Dr. Francesca D'Elia

Academic Editor

PLOS One
